# Forme pseudo-tumorale d’une tuberculose broncho-pulmonaire chez un immunocompétent mimant un cancer

**DOI:** 10.11604/pamj.2019.32.170.18129

**Published:** 2019-04-09

**Authors:** Mouhcin Daoudi, Laila Herrak, Mustapha El Ftouh, Leila Achachi

**Affiliations:** 1 Service de Pneumologie, CHU Avicenne, Rabat, Maroc

**Keywords:** Tuberculose pseudo-tumorale, pathologie néoplasique, TDM thoracique, Pseudotumoral tuberculosis, neoplasia, chest CT scan

## Abstract

La tuberculose est une maladie due à une infection par le bacille tuberculeux, tous les organes peuvent être touchés, la tuberculose pulmonaire représente un peu plus de 50% des atteintes, elle constitue un problème de santé publique dans le monde entier et particulièrement dans les pays en voie de développement. La tuberculose broncho-pulmonaire pseudo-tumorale est une forme de présentation particulière de la tuberculose chez le sujet immunocompétent, elle peut revêtir la forme d'une lésion bronchique ou pulmonaire évocatrice d'une pathologie néoplasique, que ce soit sur une TDM thoracique ou lors d'une endoscopie bronchique. Cette similitude avec la pathologie néoplasique est source de confusion pour le clinicien et rend difficile l'établissement du diagnostic positif, elle nécessite le recours à des moyens diagnostiques souvent invasifs, puisque les moyens classiques sont généralement mis à défaut, ce qui alourdit la prise en charge et retarde la mise sous traitement. L'objectif de cet article est de sensibiliser le clinicien vis-à-vis de cette forme particulière de présentation de la tuberculose, certes peu fréquente, mais à évoquer de principe devant un aspect radiologique compatible d'autant plus que le pays de résidence est de forte endémicité. Le traitement de cette forme demeure le même que celui des présentations habituelles, que ce soit en matière de médicaments ou de durée.

## Abstract (en)

Tuberculosis is a disease caused by tubercle bacillus infection. It can affect all of the body's organs. Pulmonary tuberculosis accounts for a little more than 50% of its occurrences. It is a public health problem worldwide, and developing countries in particular. Pseudotumoral bronchopulmonary tuberculosis is a particular form of tuberculosis in immunocompetent subject. It can manifests as a bronchial or pulmonary lesion, suggesting a neoplasia visible on chest CT scan or bronchoscopy. This similarity can confuse clinicians, making diagnosis challenging and requiring the use of invasive diagnostic techniques, since traditional means are often lacking. This increases burden of care and causes delayed treatment. This study aims to educate clinicians about this particular and uncommon form of tuberculosis, which should be suspected in patient with radiological findings compatible with the disease, especially since they live in a highly endemic country. Therapy and treatment duration are the same as for common forms of tuberculosis.

## Introduction

La tuberculose est une maladie qui sévit encore dans les pays en voie de développement, la forme pulmonaire est la plus fréquente des présentations, si la symptomatologie clinique est bien connue par les professionnels de santé, les aspects radiologiques sont tellement variés qu'ils peuvent parfois être trompeurs ou peu habituels et poser des problèmes diagnostiques. Parmi ces aspects peu habituels, nous avons voulu mettre la lumière sur une forme particulière qu'est la tuberculose pseudo-tumorale, qui peut prêter à confusion avec la pathologie néoplasique et qui nécessite un abord plus invasif et un temps plus long pour établir le diagnostic. Nous rapportons le cas d'un jeune patient porteur d'une masse médiastino-pulmonaire chez qui tous les arguments plaidaient pour une pathologie néoplasique, on a dû indiquer une thoracoscopie diagnostique qui, à la surprise du corps soignant et heureusement pour le patient, a permis de poser le diagnostic de tuberculose.

## Patient et observation

**Figure 1:**
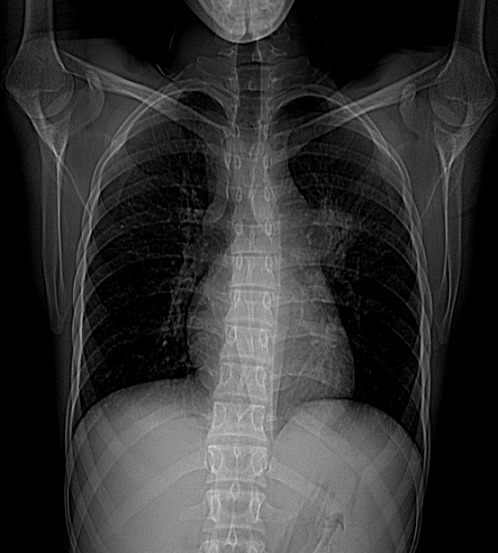
Radiographie thoracique de face montrant une opacité hilaire gauche grossièrement arrondie dont la limite interne est noyée dans l’opacité médiastinale, la limite externe relativement convexe, élargissement médiastinal

**Figure 2:**
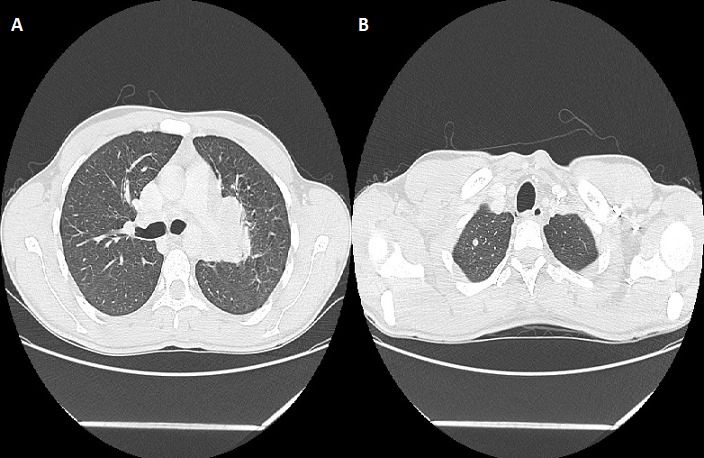
TDM thoracique: A) fenêtre parenchymateuse: masse tissulaire sus hilaire gauche mesurant 4,5 x 5cm; B) fenêtre parenchymateuse: nodule apical droit mesurant 7mm

**Figure 3:**
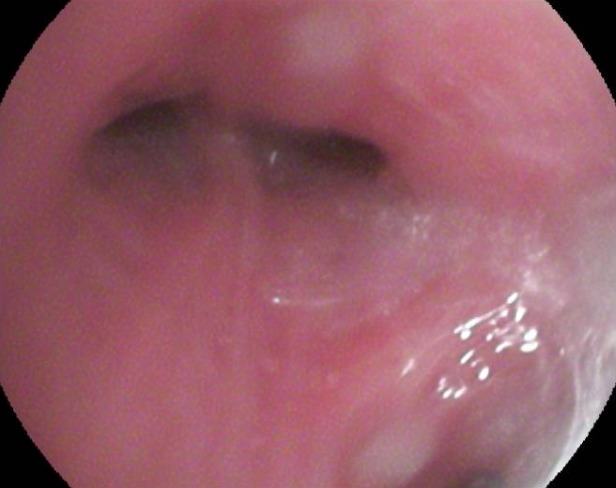
Fibroscopie bronchique: épaississement des éperons et rétrécissement des orifices de la Culmen (à gauche)

**Figure 4:**
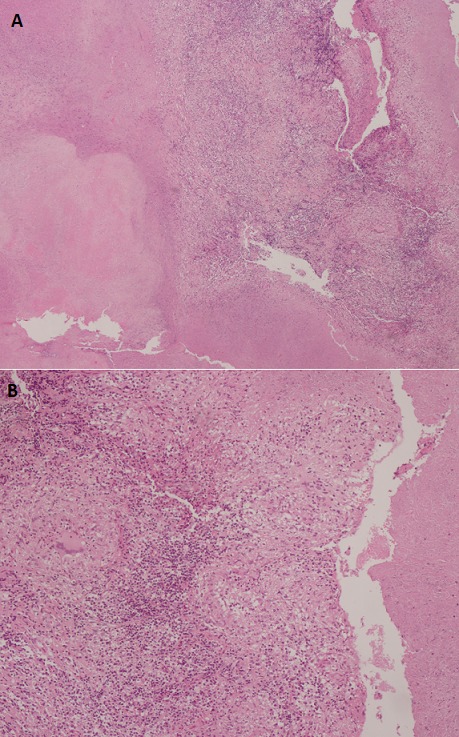
Aspect de granulome épithélio et gigantocellulaire: A) grossissement x40; B) grossissement x100

On rapporte le cas d'un jeune patient âgé de 26 ans, sans profession et sans aucune notion d'exposition, ancien tabagique à raison de 10 paquets-années sevré il y a un an, sans aucun antécédent médical ou chirurgical, n'ayant jamais été traité pour une tuberculose et sans notion de contage tuberculeux récent. La symptomatologie remontait à deux mois avant sa première consultation, faite d'une toux sèche, d'une anorexie et d'un amaigrissement non chiffré. L'examen clinique à l'admission a retrouvé un patient asthénique et amaigri (par comparaison à des photos antérieures). A noter qu'il a consulté auparavant en médecine générale et a reçu des traitements dont plusieurs cures d'antibiothérapie non spécifique sans qu'aucune amélioration clinique ou radiologique ne soit constatée. Une radiographie thoracique de face réalisée a montré une opacité hilaire gauche grossièrement arrondie, à limite interne noyée dans l'opacité médiastinale et à limite externe relativement convexe et un élargissement médiastinal (Figure 1). Un bilan biologique fait d'une NFS, crase sanguine, ionogramme était normal, la recherche de BK sur expectorations induites (ED et GeneXpert MTB/rif) était négative. Une tomodensitométrie thoracique réalisée a retrouvé une masse tissulaire hétérogène sus hilaire gauche de 4,5 X 5 cm englobant l'artère pulmonaire gauche et l'aorte thoracique sur la moitié de sa circonférence, des adénopathies médiastinales multiples de la chaine antéro-latérale gauche atteignant pour les plus volumineuses 2 cm de petit axe, un nodule de 7 mm de diamètre au niveau apical droit, le radiologue a conclu à un processus tissulaire suspect (Figure 2 A, B). Une fibroscopie bronchique a montré un aspect endoscopique normal à droite et un aspect inflammatoire de 1^er^ degré à gauche au niveau de la bronche lobaire supérieure avec un épaississement des éperons et rétrécissement des orifices des bronches segmentaires de la culmen (Figure 3), des biopsies bronchiques au niveau des éperons épaissis, une aspiration bronchique avec étude bactériologique (recherche de BK à l'examen direct et en culture, GeneXpert MTB/rif), recherche de BK dans les expectorations post-fibroscopie ont toutes été négatives. Devant la négativité du bilan réalisé, l'aspect tomodensitométrique fort évocateur d'une néoplasie, une preuve histologique s'imposait, une biopsie trans-pariétale scanno-guidée a été discutée mais récusée par les radiologues vu les contacts vasculaires étroits de la masse et le risque encouru, justifiant la réalisation d'une thoracoscopie gauche diagnostique avec biopsies ganglionnaires, l'étude histopathologique du matériel prélevé a montré l'existence de nombreux granulomes épithélio et gigantocellulaires dont certains sont centrés par de la nécrose caséeuse (Figure 4 A, B), confirmant ainsi le diagnostic de tuberculose.

## Discussion

La tuberculose broncho-pulmonaire pseudo-tumorale (TBPT) demeure une entité rare, Snene *et al*. ont colligé 1737 cas de tuberculose (toutes localisations) durant 13 ans et demi, uniquement 17 cas de TBPT ont été retenus avec une proportion de cette forme estimée à 0,97% [1], l'étude rétrospective de Chaouch *et al*. ayant porté sur 341 cas de tuberculose pulmonaire colligés sur une période de 11 ans a retenu 12 patients, avec une fréquence à 3,5% [2]. L'âge médian varie selon les publications, 40 ans en moyenne (19-64ans) d'après Snene *et al*. [1], 45 ans (20-62ans) d'après Chaouch *et al.* [2], notre patient âgé de 26 ans rejoint la médiane d'âge rapportée dans la littérature. Une intoxication tabagique est souvent retrouvée chez les patients [1-4], une prédominance masculine est notée [4], les manifestations cliniques sont non spécifiques, une toux sèche ou productive, des douleurs thoraciques, des hémoptysies et des signes généraux (fièvre, anorexie, amaigrissement) sont diversement rapportés par les patients, mais il semble que la toux et les signes généraux soient les symptômes les plus fréquents [5], tel est le cas de notre patient. Les manifestations radiologiques sont variées, il s'agit essentiellement d'opacités nodulaires ou masses, soit parenchymateuses soit hilaires, à contours irréguliers le plus souvent, d'un élargissement médiastinal en rapport avec des adénopathies, parfois des opacités rétractiles, des troubles de ventilation, ou des pleurésies peuvent se voir. La radiographie thoracique de face montre le plus souvent une opacité hétérogène à contours irréguliers, d'allure tumorale, parenchymateuse ou hilaire [5].

La TDM thoracique, peut retrouver plusieurs aspects: des condensations parenchymateuses, des masses tissulaires (hilaires ou parenchymateuses), des nodules suspects [6], un aspect de processus tumoral associé ou non à des adénopathies [7], la présentation chez notre patient était celle d'une masse tissulaire hilaire avec adénopathies. L'aspect endoscopique le plus rencontré est celui d'un bourgeon endo-bronchique et d'une sténose infiltrante [8-10], d'autres aspects moins fréquents peuvent se voir: aspect de compression extrinsèque, inflammation non spécifique, épaississement muqueux [10], aspect normal [10], notre patient avait un aspect inflammatoire avec épaississement des éperons et rétrécissement des orifices de la Culmen. La confirmation bactériologique est rarement obtenue au cours de la forme pseudo-tumorale de la tuberculose broncho-pulmonaire, ceci est dû au caractère solide et mal oxygéné des lésions caséeuses, ou la multiplication des bacilles est lente au point de leur donner un caractère quiescent , Snene *et al.* ont eu 7 cas/17 confirmés bactériologiquement [1], Chaouch *et al.* 3 cas/12 [2], Elatiqi *et al.* 4 cas/23 [4], l'étude bactériologique réalisée chez notre patient sur liquide d'aspiration bronchique (examen direct et GeneXpert MTB/rif) était négative, du liquide d'aspiration a été mis en culture sur milieu solide de Lowenstein-Jensen (culture négative après un mois). Devant la négativité du bilan et l'impossibilité d'écarter un processus tissulaire, des moyens diagnostiques plus invasifs s'imposaient, une ponction biopsie transpariétale de la masse a été discutée avec les radiologues, mais récusée car jugée risquée (contacts étroits de la masse avec les gros vaisseaux), on a du poser l'indication d'une thoracoscopie gauche diagnostique en concertation avec les chirurgiens, ce qui a permis de réaliser des biopsies ganglionnaires et une étude histologique ayant mis en évidence des granulomes épithélio et gigantocellulaires dont certains sont centrés par de la nécrose caséeuse, le diagnostic de tuberculose a été retenu. Le patient a été mis sous traitement antibacillaire selon le régime standard adopté au Maroc: 2 RHZE/4 RH, le patient a été revu après un mois de traitement, il y a eu une nette amélioration clinique, avec disparition de la toux et des signes généraux et une reprise pondérale de 4 kg, un premier contrôle radiographique est prévu à la fin de la phase initiale du traitement (après 2 mois du traitement).

## Conclusion

Il faut garder à l'esprit, particulièrement dans les pays à forte endémie de tuberculose, que toute image radiologique peut correspondre à une tuberculose, même si l'aspect radiologique n'est pas classique et qu'il est évocateur d'un autre diagnostic. Il faut s'efforcer de mettre en œuvre tous les moyens diagnostiques disponibles, invasifs ou non, à la recherche d'une preuve, soit bactériologique soit histologique, en faveur d'une tuberculose. Eviter d'annoncer rapidement des diagnostics en se basant sur une image radiologique (on avait annoncé à ce patient qu'il avait très probablement une néoplasie.) et éviter de banaliser et de traiter sans preuve comme tuberculose des patients avec des présentations radiologiques semblables sans avoir entrepris une démarche diagnostique correcte.

## Conflits d’intérêts

Les auteurs ne déclarent aucun conflit d'intérêts.
